# An Ambulance at Work

**Published:** 1887-02-26

**Authors:** 


### An Ambulance at Work.

The great Napoleon—the man of blood, of iron, and of law—was the first to conceive and originate the Modern Ambulance System. A historian of the Peninsular War says: "There was no point of warfare which more engaged Napoleon's attention than the care of his sick and wounded. Under his fostering care Larrey (justly celebrated, were it for this alone) organised the establishment called 'The Hospital Ambulance.' The astonishing rapidity with which the wounded French soldiers disappeared from a field of battle attested the excellence of this institution." The system thus originated has been gradually extending its beneficent aid to all classes in every part of the civilised world. Its latest development is the Horse Ambulance, which is now established in Liverpool. Captain Joynson, the chairman of the Northern Hospital, to whose efforts mainly the success of the system has been due on the Mersey, recently delivered a most interesting lecture to the members of the Hospitals Association. Availing ourselves of that lecture, and of other sources of information, we are able to present our readers with an account of the ambulance system as now in operation at Liverpool. "My attention," says Captain Joynson, "was first directed to the subject of ambulances during a voyage across the Atlantic at the close of 1882." The surgeon on board the Cunard ship *Gallia* described how, on his arrival at an American port, an invalid passenger, who was too weak for conveyance by ordinary methods, was removed immediately from the ship to the hospital by the ambulance without pain or danger. The picture he drew of his difficulties in Liverpool, under similar circumstances, "filled me with shame," says Captain Joynson. He was in charge of an invalid lady, who arrived at Queenstown after the voyage in a state of great prostration. He telegraphed to Liverpool for the best conveyance of an ambulance nature to be found in the town. Neither for love nor money could anything of such a description be found; and the unfortunate lady had to be stretched at full length on a mattress, and lifted on to the roof of an ordinary cab. In that way, followed by a gaping crowd of sightseers, was the poor invalid slowly drawn through the streets of the crowded city to her hotel. No wonder Captain Joynson felt ashamed of his town and country.

In 1883 the Northern Hospital resolved to try the American system. There was a difference of opinion as to charging the cost of the department in the ordinary accounts of the hospital. It was, therefore, determined to make it a *separate charity*, to be met by a *separate subscription list*. Captain Joynson insists upon two things as of the first importance in an ambulance system. The first is—*police co-operation;* and the second—*rapid means of communication*. In Liverpool there has been no difficulty on either of these points. The police have found that it is much easier to send for the ambulance than to carry accidents and other cases through street after street on stretchers. For rapid communication, a wire from the hospital to the telephone centre, and a chart showing the whereabouts of all the subscribers to the telephone system, enable messages to be sent to the police station and to the hospital within a few minutes at the longest. The subscribers have always shown the utmost readiness to give all the help in their power.

Captain Joynson gives an account of his experience at the ambulance station in Chicago. "I was taken," he says, "into a square and roomy coachhouse, a carriage being stationed in the centre with some patent harness swinging from the ceiling. Two attendants were chatting in one corner of the room, and a horse was champing his hay in a railed-off box in the other. .. The electric bell rang. The men sprang to their feet; the horse, with a start and a whinney, pushed the door of the box open with his head, and took his place near the shafts of the vehicle. To lower the harness on to his back and hitch up, was the work of *a few seconds*, and so the carriage was ready for the road."

What is an ambulance? Many people think of it as merely a large comfortable carriage with a bed in it. But, as a matter of fact, that is but a small part of it. When properly constructed and complete, it is a *Moving Hospital*, with all the paraphernalia of a hospital; and no sooner is the patient put to bed inside than the surgeon can begin operations, and get the fracture temporarily put up, or the dislocation reduced, before the hospital gates are in sight. What does this mean to a man with a bad fracture or other dangerous accident? It often means hours of freedom from agonising and indescribable pain; and not seldom it means the saving of life itself.

A short description of the ambulance carriage now in use at the Liverpool Northern Hospital will be here appropriate. The carriage was designed by Mr. John Furley, of the St. John's Ambulance Association. It is built of English oak, and American ash and birch. There is room inside for two patients and three attendants, with stretcher beds for the former and seats for the latter. Generally the carriage is required for one patient only at a time, the stretcher being placed upon the floor. A second stretcher can be put on a shelf, which may also be used as a seat. One stretcher is padded and jointed, in order that it may adapt itself readily to the posture easiest for the patient; the other is of the Furley pattern, and folded. Both are provided with telescopic handles. There is a box inside for medical appliances; and the driver's seat, besides accommodating two persons, is also used as a receptacle for bandages, splints, and a variety of other medical and surgical requisites. When an accident occurs, two moves only are required by the patient,—viz., from the ground to the stretcher, and from the stretcher to the bed in the hospital. How many movements may be inevitable when a cab does duty instead of an ambulance, it is impossible to calculate.

It is generally thought that the poor only are benefited by these conveniences for easy transit; but the experience of Liverpool shows that very frequent use is made of the ambulance by the better classes. It is never refused when an application is made, although it is usual for a small sum to be demanded, according to the position of the applicant. The comfort to the patient is found to be so great, that very often handsome contributions are made to the funds on these occasions.

*The cost* of such a system as we have described is a question of much practical importance, and one about which there are many erroneous notions. We therefore give some details here of the actual expense incurred at Liverpool for twelve months' work. Of course, much will depend upon the character of the stable-accommodation which is started with. It will probably be found on the whole better to hire than to keep horses. Some risks and annoyances are avoided, and there is usually no difficulty in hiring horses on such terms as to secure that they shall be at all times promptly available.

EXPENSES OF AMBULANCE FOR ONE YEAR.

| | £ | s. | d. |
|---|---|---|---|
| Driver's wages at 24s. per week.. .. .. | 62 | 8 | 0 |
| Horse hire at 30s. „ .. .. .. | 78 | 0 | 0 |
| Rent of stable .. .. .. .. .. .. | 20 | 0 | 0 |
| Cottage for driver at 6s. per week .. .. | 15 | 12 | 0 |
| Livery for driver .. .. .. .. .. | 6 | 6 | 0 |
| Telephone (private wire) .. .. .. .. | 15 | 10 | 0 |
| Insurance .. .. .. .. .. .. | 4 | 10 | 0 |
| Repairs and sundries .. .. .. .. | 25 | 0 | 0 |
| | 227 | 6 | 0 |

The actual cost per annum after the initial outlay is thus seen to be very small. In London and other large centres of population, where there are more hospitals than one, the same ambulance might serve for three or four; and this, with the sums paid for its occasional use by the better classes, would still further reduce the cost to each hospital.

This account of an ambulance system in actual operation should prove of interest to everyone whose business or pleasure leads him daily to traverse the streets of a large town, or to undertake frequent journeys by rail. Who can tell which of us it next may be who will suffer agonies for want of such an ambulance, or who will be grateful to those whose efforts have brought one to our aid? For Londoners especially this is a question which comes immediately home to every one. For here most of the large hospitals are crowded together within a very narrow area, many of them being not more than a mile and a half from Charing Cross. Persons injured in remote and outlying districts are often hours before they reach the hospital, and by that time the patient in many instances is more dead than alive with prolonged agony and loss of blood. Yet in London there is no ambulance at all for the general hospitals.

Since July 1883 the Liverpool Ambulance Service has carried no fewer than 2,033 cases, 647 being thus conveyed last year alone. Of these 6 were spinal fractures (broken backs), 24 fractures of the ribs and pelvis, 22 compound and 91 simple fractures of the extremities. But small powers of imagination are needed, especially by those who have once witnessed a street or a railway accident, to enable them to realise the incalculable amount of unnecessary suffering and danger which have thus, by a little cost and trouble, been entirely avoided.

